# Lack of Interferon (IFN) Regulatory Factor 8 Associated with Restricted IFN-γ Response Augmented Japanese Encephalitis Virus Replication in the Mouse Brain

**DOI:** 10.1128/JVI.00406-21

**Published:** 2021-10-13

**Authors:** Aarti Tripathi, Bhupendra Singh Rawat, Sankar Addya, Milan Surjit, Prafullakumar Tailor, Sudhanshu Vrati, Arup Banerjee

**Affiliations:** a Translational Health Science and Technology Institute (THSTI), Faridabad, India; b National Institute of Immunology (NII), New Delhi, India; c Regional Center for Biotechnology (RCB), Faridabad, India; d Sidney Kimmel Cancer Center, Thomas Jefferson University, Philadelphia, Pennsylvania, USA; Hudson Institute of Medical Research

**Keywords:** IRF8, microglial activation, IFN-γ, neuroinflammation, Japanese encephalitis virus, interferons, microglia

## Abstract

Interferon regulatory factor 8 (IRF8), a myeloid lineage transcription factor, emerges as an essential regulator for microglial activation. However, the precise role of IRF8 during Japanese encephalitis virus (JEV) infection in the brain remains elusive. Here, we report that JEV infection enhances IRF8 expression in the infected mouse brain. Comparative transcriptional profiling of whole-brain RNA analysis and validation by quantitative reverse transcription-PCR (qRT-PCR) reveals an impaired interferon gamma (IFN-γ) and related gene expression in *Irf8* knockout (*Irf8^−/−^*)-infected mice. Further, *Ifnγ* knockout (*Ifnγ*^−/−^) mice exhibit a reduced level of *Irf8.* Both *Ifnγ*^−/−^ and *Irf8^−/−^* mice exhibit significantly reduced levels of activated (CD11b^+^ CD45^hi^, CD11b^+^ CD45^lo^, Cd68, and CD86) and infiltrating immune cells (Ly6C^+^, CD4, and CD8) in the infected brain compared to those of wild-type (WT) mice. However, a higher level of granulocyte cell (Ly6G^+^) infiltration is evident in *Irf8^−/−^* mice as well as the increased concentration of tumor necrosis factor alpha (TNF-α), interleukin-6 (IL-6), monocyte chemoattractant protein 1 (MCP1) levels in the brain. Interestingly, neither the *Irf8^−/−^* nor the *Ifnγ*^−/−^ conferred protection against lethal JEV challenge to mice and exhibit augmentation in JEV replication in the brain. The gain of function of *Irf8* by overexpressing functional IRF8 in an IRF8-deficient cell line attenuates viral replication and enhances IFN-γ production. Overall, we summarize that in the murine model of JEV encephalitis, IRF8 modulation affects JEV replication. We also show that lack of Irf8 affects immune cell abundance in circulation and the infected brain, leading to a reduction in IFN-γ level and increased viral load in the brain.

**IMPORTANCE** Microglial cells, the resident macrophages in the brain, play a vital role in Japanese encephalitis virus (JEV) pathogenesis. The deregulated activity of microglia can be lethal for the brain. Therefore, it is crucial to understand the regulators that drive microglia phenotype changes and induce inflammation in the brain. Interferon regulatory factor 8 (IRF8) is a myeloid lineage transcription factor involved in microglial activation. However, the impact of IRF8 modulation on JEV replication remains elusive. Moreover, the pathways regulated by IRF8 to initiate and amplify pathological neuroinflammation are not well understood. Here, we demonstrated the effect of IRF8 modulation on JEV replication, microglial activation, and immune cells infiltration in the brain.

## INTRODUCTION

Japanese encephalitis virus (JEV) is a neurotropic virus and the leading cause of viral encephalitis in Asia. About 70,000 cases are reported each year, of which approximately 20% to 30% are fatal (1). The patients who recover from infection develop severe neurological and psychiatric sequelae. Microglial cells, the resident macrophages in the brain, play a vital role in JEV pathogenesis. Microglia become activated in response to JEV infection, enhancing the prolonged release of proinflammatory mediators in the brain (reviewed in reference 2). It is now evident that microglia can have both neuroprotective and neurotoxic effects. The deregulated activity of microglia can be lethal for the brain. Several reports indicated that activated microglia may damage or induce the apoptotic death of neurons, directly releasing toxic mediators such as cytokines and free radicals or indirectly attracting activated T cells, monocytes, and neutrophils into the central nervous system (CNS) (3). Therefore, it is critical to understand the mediators that drive changes in microglia phenotypes during JEV infection.

Recently, interferon regulatory factor 8 (IRF8), a myeloid lineage transcription factor, was identified as a critical molecule for microglial activation (4). IRF8 was also extensively studied for its functional significance in innate and adaptive immune response (5, 6). IRF8 is a transcription factor expressed in immune cells, such as lymphocytes, dendritic cells, macrophages, and microglial cells. IRF8 may activate a set of gene expressions that transforms microglia into a reactive phenotype (7). Overexpression of IRF8 in microglial cells helps microglia reach a proinflammatory-reactive state. Simultaneously, deficiency of IRF8 in mice conferred them resistance to peripheral nerve injury (4) and experimental cerebral malaria (8). IRFs have a highly conserved DNA binding domain (120 amino acids). The N termini of the domain recognize the interferon (IFN)-stimulated response element (ISRE) DNA motif. In contrast, the variable IRF association domain (IAD) determines the binding affinity to other protein partner molecules acting as a regulatory domain (9).

Previous studies have revealed the role of IRFs in the development, differentiation, and function of the immune cell (9). Irf8^R294C^ mutant mice showed monocyte polarization to granulocyte lineage (neutrophils) with chronic myeloid leukemia (CML) syndrome in BXH2 mice. BXH2 mice are also susceptible to vesicular stomatitis virus (VSV) infection and generate an inadequate antiviral response in *Irf8* mutant mice (10). Horiuchi et al. reported that IRF8-deficient microglia are not phenotypically and functionally equivalent to wild-type microglia, as they were less capable of sensing tissue damage due to their less extended processes (11). So, IRF8 regulates the morphology of microglia during homeostasis and inflammatory condition. A subsequent study by Berghout et al. demonstrated severe loss of IRF8 function in BXH2 mice that completely protects the animals against cerebral malaria, preventing neurological symptoms with prolonged postinfection survival of BXH2 mice (8). Evidence from a genome-wide association study (GWAS) in humans has shown an association of IRF8 variants to chronic inflammatory conditions, such as multiple sclerosis, cerebral malaria, and peripheral nerve injury (12). Xu et al. reported that NOTCH-RBP-J signaling regulates the transcription factor Irf8 to promote inflammatory macrophage polarization (13). A recent study suggested that IRF8 can stimulate interleukin-12 (IL-12) p40 and IL-23 production but inhibits IL-27 (14). Inhibition of IL-27, in turn, increases the intra-CNS amplification of Th1 and Th17 cells, leading to microglial activation and accelerates neuroinflammation. Together, these studies infer the crucial role of IRF8 in microglial differentiation, activation, and neuroinflammation.

Our previous study reported increased IRF8 expression in JEV-infected human microglial cells (15). IRF8 plays a vital role in the physiology of both peripheral and microglial cells; yet, its role in immune modulation in the context of JEV infection remains unknown. Here, we conducted a study to understand the effect of IRF8 on microglial activation and JEV replication in the brain. We have used an experimental model of murine encephalitis induced by infection with JEV to investigate the role of IRF8 in pathological inflammation. This model used 3- to 4-week-old C57BL/6 mice infected with JEV (P20778 JEV-S3 strain) through the intraperitoneal (i.p.) route. Clinical signs such as piloerection, body stiffening, restricted movement, tremor, and hind limb paralysis appeared between day 5 and day 8 postinfection (p.i.) in susceptible mice, progressing to death within 24 to 48 h postonset of clinical signs. Using IRF8 knockout (KO) mice, we demonstrated the effect of IRF8 modulation on JEV replication. We also provide evidence that the lack of Irf8 affects immune cell abundance in circulation and the brains of infected mice.

## RESULTS

### JEV infection induces IRF8 expression in infected mouse brain and microglia.

**FIG 1:**
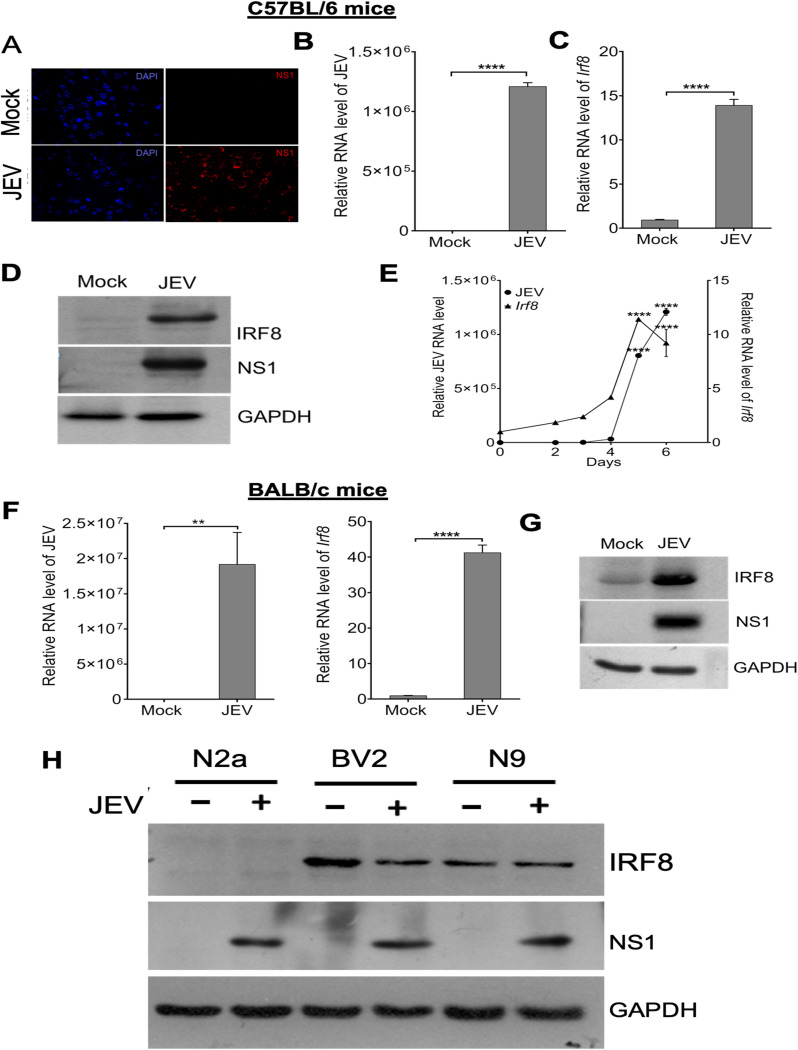
Interferon regulatory factor 8 (IRF8) expression increases upon JEV infection in mouse brains. Three-weeks-old (C57BL/6) mice (*n* = 3 mice/group) were administered with JEV, p20778 strain (10^7^pfu), or minimal Eagle’s medium (mock) through the intraperitoneal route. Brains were harvested 24 h postonset of encephalitic symptoms. The viral and IRF8 proteins and RNA were detected by immunofluorescence, Western blotting, and qRT-PCR method. (A) Brain sections were stained for NS1 (red) and 4′,6-diamidino-2-phenylindole (DAPI) (blue). (B, C) Relative JEV RNA level and *Irf8* transcript level were quantified by qRT-PCR (normalized to *Gapdh* mRNA; ****, *P* < 0.0001). (D) Total protein from brain homogenate was Western blotted for NS1, IRF8, and GAPDH detection. (E) Viral RNA and IRF8 transcript expressions were measured at six different time points (0, 2, 3, 4, 5, 6 days postinfection [dpi]) in the brains of mock and infected mice (normalized to *Gapdh* mRNA; ****, *P* < 0.0001). BALB/c mice (p-10, *n* = 3 mice/group) were infected either with JEV, GP78 strain (10^5^ PFU), or with 1× PBS (mock), and 24 h postonset of encephalitic signs, brains were harvested to prepare RNA and protein. (F) Relative JEV RNA level and Irf8 transcript expression in the mock- and JEV-infected mice analyzed by quantitative RT-PCR normalized to Gapdh mRNA). **, *P* < 0.01; ****, *P* < 0.0001. (G) Total protein from brain homogenate was Western blotted for NS1, IRF8, and GAPDH detection. (H) Mouse neuronal cells (N2a) and microglia cell lines (BV2 and N9) were mock or JEV infected at an MOI of 5 and harvested 24 hours postinfection (h p.i.). Cell lysates were immunoblotted for IRF8 and GAPDH expression. All qRT-PCR data are represented as mean ± SD.

To understand JEV infection’s effect on IRF8 expression, we infected 3- to 4-week-old C57BL/6 mice with the JEV P20778 strain through the i.p. route. The JEV NS1 protein was detected in the infected brain by immunofluorescence and Western blotting assay. JEV infection was evident in infected mice, as viral protein and RNA were detected in the brain, indicating that the virus reached the brain (Fig. 1A and B). Further, the IRF8 level was significantly enhanced in infected mouse brains (Fig. 1C and D). Upon JEV infection through the i.p. route, viral RNA was detectable in the brain as early as day 2 (p.i.) (Fig. 1E), and viral RNA level increased as the symptoms developed at day 5 p.i. We also observed that the *Irf8* transcript was significantly increased in a time-dependent manner (Fig. 1E) and correlated with virus RNA levels in the brain.

To nullify that virus-induced IRF8 upregulation is not a strain-specific effect, we verified IRF8 expression in BALB/c mice infected with JEV strain GP78. We observed increased IRF8 expression along with viral RNA and protein in the infected brain, suggesting IRF8 upregulation is not a strain-specific phenomenon; instead, it is a universal phenomenon (Fig. 1F and G).

Apart from neuronal cells, JEV also infects microglial cells. Therefore, it is essential to understand which cells are contributing to IRF8 upregulation in the infected brain. We infected mouse neuroblastoma (N2a) as well as microglia (BV2 and N9) cells. We checked IRF8 expression in the mock and infected cells. We observed that IRF8 did not express in neuronal cells but constitutively expressed in microglial cells (Fig. 1H).

### IRF8 deficiency affects the expression of microglia/macrophage activation genes and modulates cytokine production in the JEV-infected mouse brain.

**FIG 2:**
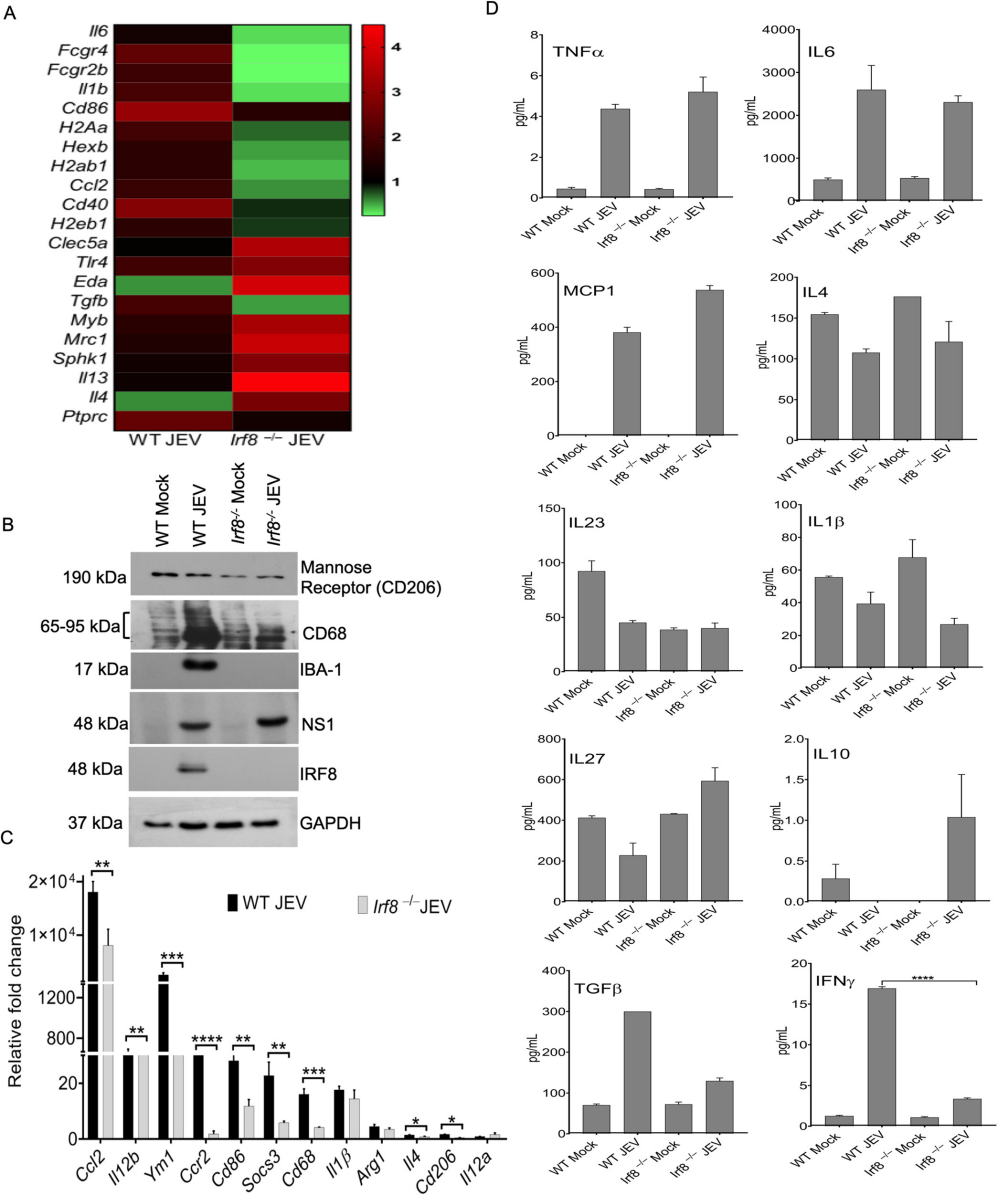
Effect of Irf8 deficiency on microglia and infiltrating macrophage phenotype. Total RNA was isolated from the WT and *Irf8^−/−^* mouse brains of either mock- or JEV-infected groups at 24 h postonset of symptoms to evaluate the gene expression. (A) The heat map represents different microglia marker expression levels between WT and *Irf8*^−/−^ infected mice obtained from the qPCR array. (B) Representative Western blotting data show the status of different activation markers for microglia and infiltrated macrophages. (C) qRT-PCR was performed for quantitation of relative levels of indicated genes. *Gapdh* mRNA level was used for normalization. All qRT-PCR data are represented as mean ± SD. *, *P* < 0.05; **, *P* < 0.01; ***, *P* < 0.001; ****, *P* < 0.0001. (D) Cytokine expression was quantified in the protein lysates of WT and *Irf8^−/−^* mouse brains of either mock- or JEV-infected groups using cytokine bead array kits analyzed by FCAP and Qognit software. Representative data are shown; ****, *P* < 0.0001.

IRF8 is involved in the proinflammatory M1 phenotype’s commitment to microglia and macrophages (4, 11, 13). During JEV infection, it is evident that microglia become activated in the nervous system and play an essential role in neuronal pathologies. The transcription factor, IRF8, acts as a critical regulator of reactive microglia and activates a gene expression program that transforms microglia into a reactive phenotype (4). JEV infection also induces cytokine expression in infected microglial cells. To assess the effect of IRF8 on M1 and M2 marker gene expression and subsequent proinflammatory cytokine production in the infected brain, we performed a quantitative reverse transcription-PCR (qRT-PCR) array and cytokine bead assay of the RNA and protein, respectively, from mock/infected wild-type (WT) and *Irf8^−/−^* mice in the brain. We observed that most of the M1 genes (Il6, Fcgr4, Fcgr2b, Il1b, Cd86, H2Aa, Hexb, and Ccl2) were downregulated, while M2 genes (e.g., Eda, Myb, Mrc1, Sphk1, Il3, and Il4) were upregulated (Fig. 2A). Additionally, we checked the Mannose receptor, Iba1, CD68 markers at the protein level. Compared to WT JEV-infected mice, all of the markers showed significant reductions in *Irf8^−/−^* mice (Fig. 2B). We further validated additional inflammatory M1 markers, e.g., *Ccl2*, *Ccr2*, *Cd68*, *Cd86*, *Ym1*, *Socs3*, *Il12b.* Their levels were significantly increased in WT mice but attenuated in *Irf8^−/−^* mice, suggesting microglia and infiltered macrophages are less responsive toward proinflammatory response in *Irf8^−/−^* mice. Conversely, *Ym1* was highly downregulated, while no significant changes in *Arg1*, an M2, or anti-inflammatory markers were observed in WT and *Irf8^−/−^* mice (Fig. 2C). JEV pathophysiology is associated with inflammatory cytokine release (16), influencing the disease's course and the outcome. Hence, we measured tumor necrosis factor alpha (TNF-α), IL-6, monocyte chemoattractant protein 1 (MCP1), IL-4, IL-23, and IL-1β at the protein level. We found a significant increase in the level of three cytokines (TNF-α, IL-6, and MCP1) and a decrease in the IL-4 and IL-1β levels in the WT and *Irf8^−/−^* infected brain compared to mock-infected mice (Fig. 2D). IL-27 is a member of the IL-6/IL-12 family and a pleiotropic cytokine playing an essential role in immunomodulation, as it suppresses inflammation by inhibiting Th17 response (14, 17) and promotes IL-10 production (18). We observed increased IL-27 and IL-10 levels in infected *Irf8^−/−^* mice compared to those of WT infected mice. Interestingly, we observed highly suppressed transforming growth factor β (TGF-β) and IFN-γ in infected *Irf8^−/−^* mice compared to those in WT mice (Fig. 2D). These results suggest that the presence of IRF8 is essential for the expression of microglia/macrophage activation genes and modulates JEV-induced proinflammatory cytokine production in the infected brain.

### IRF8 affects interferon genes and attenuates interferon-stimulated gene expression in JEV-infected mouse brain.

**FIG 3:**
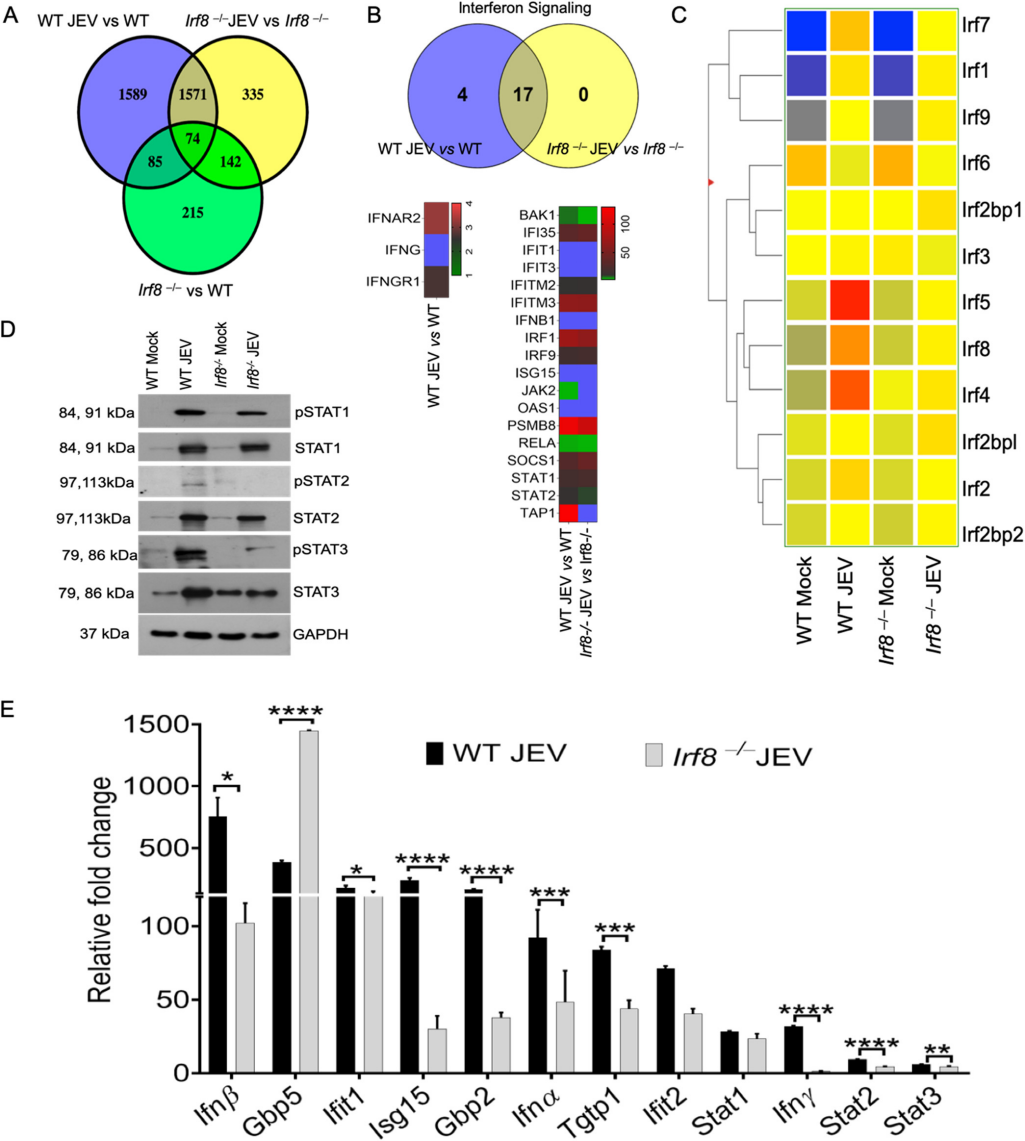
Transcriptome profiling to elucidate genes significantly affected in WT and *Irf8^−/−^* mouse brains following JEV infection analyzed by RNA-Seq. To reveal the global transcriptional changes in the whole brain, RNA sequencing of RNA samples isolated from WT and *Irf8^−/−^* mouse brains of either mock- or JEV-infected groups was performed. (A) Venn diagram showing differentially expressed genes generated by a comparative analysis of WT JEV versus WT, *Irf8^−/−^* JEV versus *Irf8 ^−/−^*, and *Irf8^−/−^* versus WT groups. (B) Venn diagram and heat map plot of genes associated with the interferon signaling pathway identified using Metascape pathway enrichment analysis of infected versus uninfected WT and *Irf8^−/−^* transcript profiles. Blue represented higher expression values. (C) Clustered heat map analysis of interferon regulatory factors extracted from infected versus uninfected WT and *Irf8^−/−^* transcript profiles. (D) Representative Western blotting data showing the status of different STATs. (E) mRNA expression of interferon-stimulated genes identified during RNA sequencing analysis was validated in the mock/infected WT and *Irf8^−/−^* mouse brains, analyzed by quantitative RT-PCR (normalized to Gapdh mRNA). All qRT-PCR data are represented as mean ± SD; *, *P* < 0.05; **, *P* < 0.01; ***, *P* < 0.001; ****, *P* < 0.0001.

IRF8 acts as a positive regulator of IFN and proinflammatory signaling in the murine system. Cooperative interaction of IRF8 and other interferon regulatory factors (IRFs), e.g., IRF3, IRF7, IRF5, and IRF4, can modulate type I and type II interferon-producing myeloid lineage cells (19, 20). Sufficient production of interferons and antiviral genes is crucial for mounting an effective immune response against invading pathogens. To understand how IRF8 modulation affects interferon gene expressions and antiviral genes, we performed high-throughput RNA sequencing (RNA-Seq) on whole-brain samples isolated from mock- or JEV-infected WT and *Irf8^−/−^* mice. The Venn diagram showed the common and unique genes detected in WT and *Irf8^−/−^* infected mice (Fig. 3A). We observed 17 dysregulated interferon genes shared in the WT and *Irf8^−/−^* infected mouse brains. However, their expression levels varied (Fig. 3B). Importantly, *Ifnγ* was detected only in WT infected mice but not in *Irf8^−/−^* mice. When we looked at different IRFs, we found that *Irf7*, *Irf8*, *Irf5*, and *Irf4* were significantly upregulated in WT mouse brains upon infection with JEV. However, these IRFs were reduced in *Irf8^−/−^* mice (Fig. 3C).

IRFs drive downstream interferon gene expressions via activation of the signal transducer and activator of transcriptions (STATs). Therefore, we looked into various STATs in WT and *Irf8^−/−^* infected mice. We observed a significant reduction of phospho STAT1, 2, and 3 in *Irf8^−/−^* mice compared to those in WT infected mice (Fig. 3D). We further checked different interferon-stimulated genes expressions. The *Ifnα*, *Ifnβ*, and *Ifnγ* levels were significantly reduced in *Irf8^−/−^* mice upon JEV infection. Not only that, except for *Gbp5*, all of the other *Isgs* (e.g., *Ifit1*, *Ifit2*, *Gbp2*, *Isg15*, *Tgtp1*) levels also decreased significantly in *Irf8^−/−^* mice (Fig. 3E). These results suggest that the absence of IRF8 limits antiviral response against JEV infection in mice.

### Interferon-gamma knockout mice exhibited a reduced level of IRF8 and increased viral load in the infected brain.

**FIG 4:**
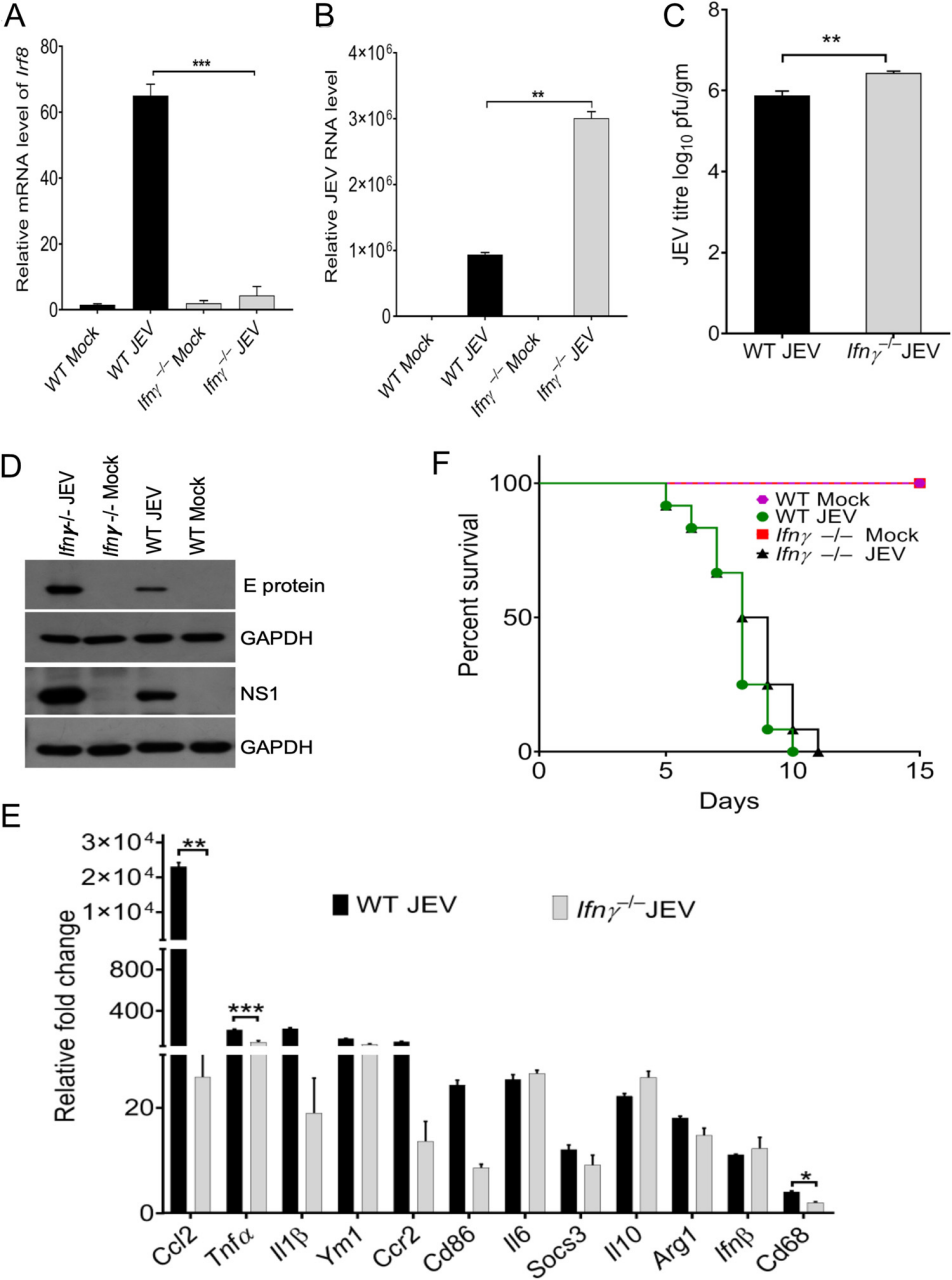
Effect of *Ifnγ* deficiency on viral load and activation genes of microglia and infiltrated macrophage. Total RNA was isolated from the WT and *Ifnγ^−/−^* mouse brains of mock- or JEV-infected groups at 24 h postonset of clinical signs. (A, B) The relative RNA level of Irf8 and JEV transcripts were quantified by qRT- PCR in WT (C57BL/6) and Ifnγ^−/−^ mouse brain RNA (normalized to *Gapdh* mRNA). ***, *P* < 0.001; **, *P* < 0.01. (C) Virus titers in the brain homogenates of infected WT and *Ifnγ^−/−^* mouse brains were determined through plaque assay on the PS cell line. Virus titer is expressed as the number of virion particles per gram; **, *P* < 0.01. (D) Total protein in the indicated groups was Western blotted for E protein, NS1, and GAPDH detection. (E) qRT-PCR was performed for quantitation of relative levels of indicated genes (normalized to Gapdh mRNA). Representative data are shown. All qRT-PCR data are represented as mean ± SD; *, *P* < 0.05; **, *P* < 0.01; ***, *P* < 0.001. (F) A survival study was performed to assess the survival of *Ifnγ^−/−^* mice to JEV infection. WT (C57BL/6) and *Ifnγ^−/−^* mice (*n* = 12 in each group) were mock and JEV (10^7^ PFU) infected, followed by the appearance of encephalitic symptoms until death; ****, *P* < 0.0001. The data shown are the combined results of three experiments.

From our RNA-Seq data analysis, it is evident that IFN-γ appeared to be associated with IRF8 expression and was severely compromised in *Irf8^−/−^* mice. To understand that IRF8-mediated effects were possibly associated with IFN-γ expression, we infected *Ifnγ^−/−^* mice with JEV and monitored them. Interestingly, when we checked the IRF8 expression in the infected *Ifnγ^−/−^* mice, we observed a significant reduction in the *Irf8* in knockout mice compared to that in WT mice at the transcript level (Fig. 4A). Moreover, JEV replication was significantly enhanced in *Ifnγ^−/−^* mice (Fig. 4B to D). Interestingly, survival curve analysis suggested that *Ifnγ^−/−^* mice developed symptoms earlier than WT mice upon viral infection, but no significant changes were observed in survival time (Fig. 4F). Like *Irf8^−/−^* mice, *Ifnγ^−/−^* mice exhibited lower expression of *Ccl2*, *Cd68*, *Cd86*, and *Ym1*, highlighting reduced activation of microglia and infiltrating macrophages in the brain (Fig. 4E).

### Lack of *Ifnγ* and *Irf8* modulate immune cell abundance in circulation and affect immune cell infiltration in JEV-infected brain.

**FIG 5:**
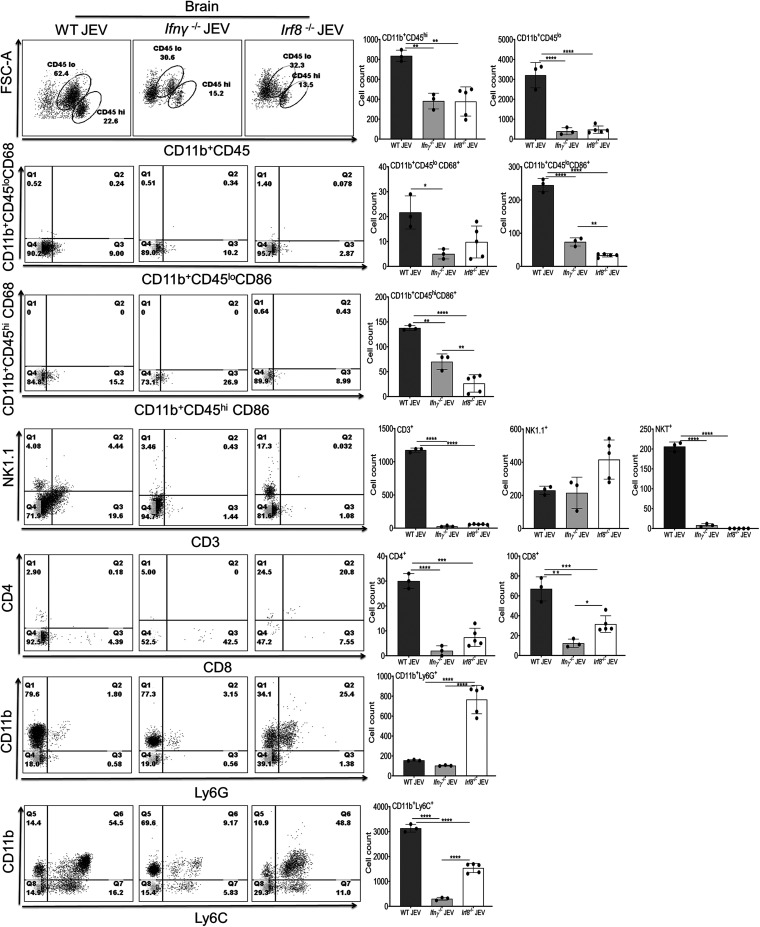
Immune cells infiltration into the brain 24 h postonset of clinical signs in WT, *Ifnγ^−/−^*, and *Irf8^−/−^* mice. Myeloid and lymphoid cells were enriched from the brains of WT (*n* = 3), *Ifnγ****^−/−^*** (*n* = 3), and *Irf8^−/−^* (*n* = 5) mice either of mock- or JEV-infected groups, by Percoll gradient for the flow cytometric analysis. Representative dot plots (left) show percentage of cells in each quadrant; positive for the indicated markers on the axis and bar graph (right panel) indicates the quantification of CD45^+^, CD11b^+^, CD68^+^, CD86^+^, Ly6C^+^, Ly6G^+^, CD3^+^, CD4^+^, CD8^+^, and NK1.1^+^ expression on leukocytes (**, *P* < 0.01; ***, *P* < 0.001; ****, *P* < 0.0001). Each dot represents cells from an individual mouse. Error bars are represented as mean ± SD.

To check whether the lack of *Ifnγ* and *Irf8* had any impact on the activation of microglia or infiltrating immune cells in the infected brain, we harvested mouse brains from WT, *Ifnγ^−/−^*, and *Irf8****^−/−^*** infected mice 24 h postonset of clinical signs and analyzed the influx of immune cells in the brain. Our fluorescence-activated cell sorter (FACS) data (Fig. 5) show that activated macrophage (CD11b^+^ CD45^hi^) and microglia (CD11b^+^ CD45^lo^) cells were significantly higher in the WT than in the *Ifnγ^−/−^* and *Irf8^−/−^* infected mice. Moreover, CD68 and CD86 marker (representing M1 microglia/macrophage activation) expressions were significantly reduced in *Ifnγ^−/−^* and *Irf8^−/−^* infected mice. Interestingly, we did not observe significant differences in NK1.1 levels in brains of WT, *Ifnγ^−/−^*, or *Irf8^−/−^* infected mice, but the NKT cell count was higher in WT mice.

**FIG 6:**
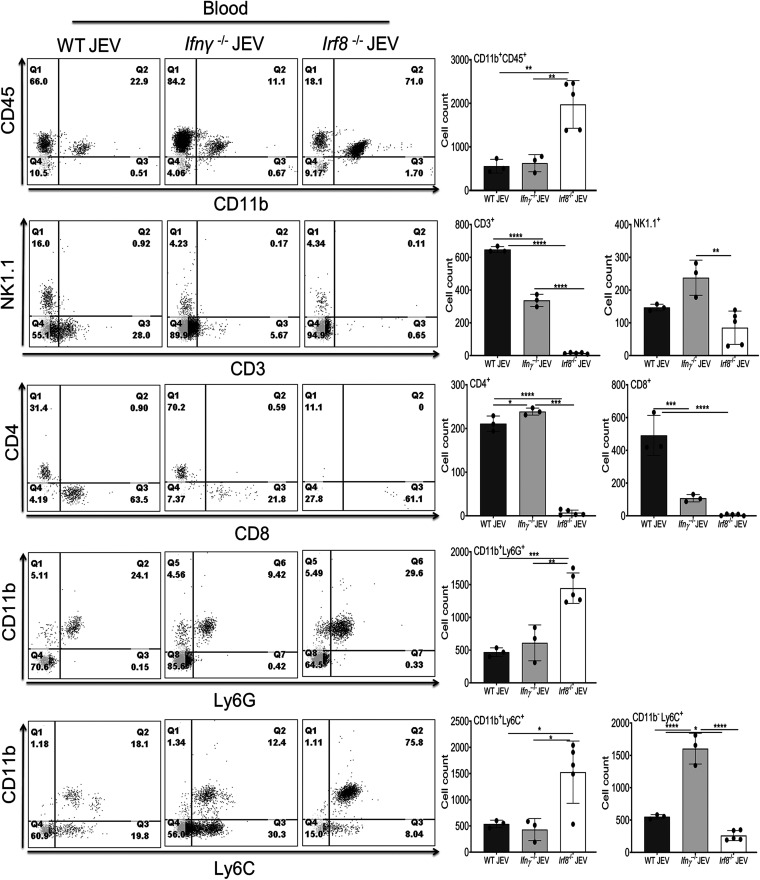
Immune cell abundance in blood 24 h postonset of clinical signs in WT, *Ifnγ^−/−^*, and *Irf8^−/−^* mice. Leukocytes from the blood of WT (*n* = 3), *Ifnγ****^−/−^*** (*n* = 3), and *Irf8^−/−^* (*n* = 5) mice, either of mock- or JEV-infected groups, were subjected to flow cytometric analysis. Representative dot plot (left) shows the percentage of cells in each quadrant, positive for the indicated markers on the axis. Bar graph (right) indicates the quantification of CD45^+^, CD11b^+^, Ly6C^+^, Ly6G^+^, CD3^+^, CD4^+^, CD8^+^, and NK1.1^+^ expression on leukocytes (**, *P* < 0.01; ***, *P* < 0.001; ****, *P* < 0.0001). Each dot in the bar graph represents cells from an individual mouse. Error bar are represented as mean ± SD.

Compared to WT mice, CD4 and CD8 T cells were less abundant in *Irf8^−/−^* infected brains and almost undetectable in *Ifnγ^−/−^* infected mice. We did not observe significant changes in Ly6G^+^ cells in WT and *Ifnγ^−/−^* infected mice, but 4- to 5-fold more infiltration of Ly6G^+^ cells was observed in *Irf8^−/−^* infected brains. Simultaneously, CD11b^+^ Ly6C^+^ cells representing the monocytes were highly abundant in WT mice compared to those in *Ifnγ^−/−^* and *Irf8^−/−^* infected mice. Interestingly, we did not see much difference in CD11b^+^ CD45^+^, Ly6C^+^, and CD4^+^ populations in peripheral blood between WT and *Ifnγ^−/−^* mice (Fig. 6). However, we did notice an increased accumulation of CD11b^−^ Ly6C^+^, representing myeloid progenitor cells in the blood of *Ifnγ^−/−^* infected mice (Fig. 6) but failing to migrate to the brain (Fig. 5). In contrast, *Irf8^−/−^* mice exhibited a higher abundance of CD11b^+^ CD45^+^, Ly6G^+^, and Ly6C^+^ cells in the blood. Apart from this, CD4 and CD8 cells were less abundant in *Irf8^−/−^* infected mice (Fig. 6).

Thus, our data suggest that the lack of IFN-γ reduced IRF8 expression, and the absence of *Irf8* or *Ifnγ* modulates the abundance of immune cells in the blood and brain. Moreover, lack of *Ifnγ* and IRF8 probably affects immune cell infiltration and activation in response to JEV infection.

**FIG 7:**
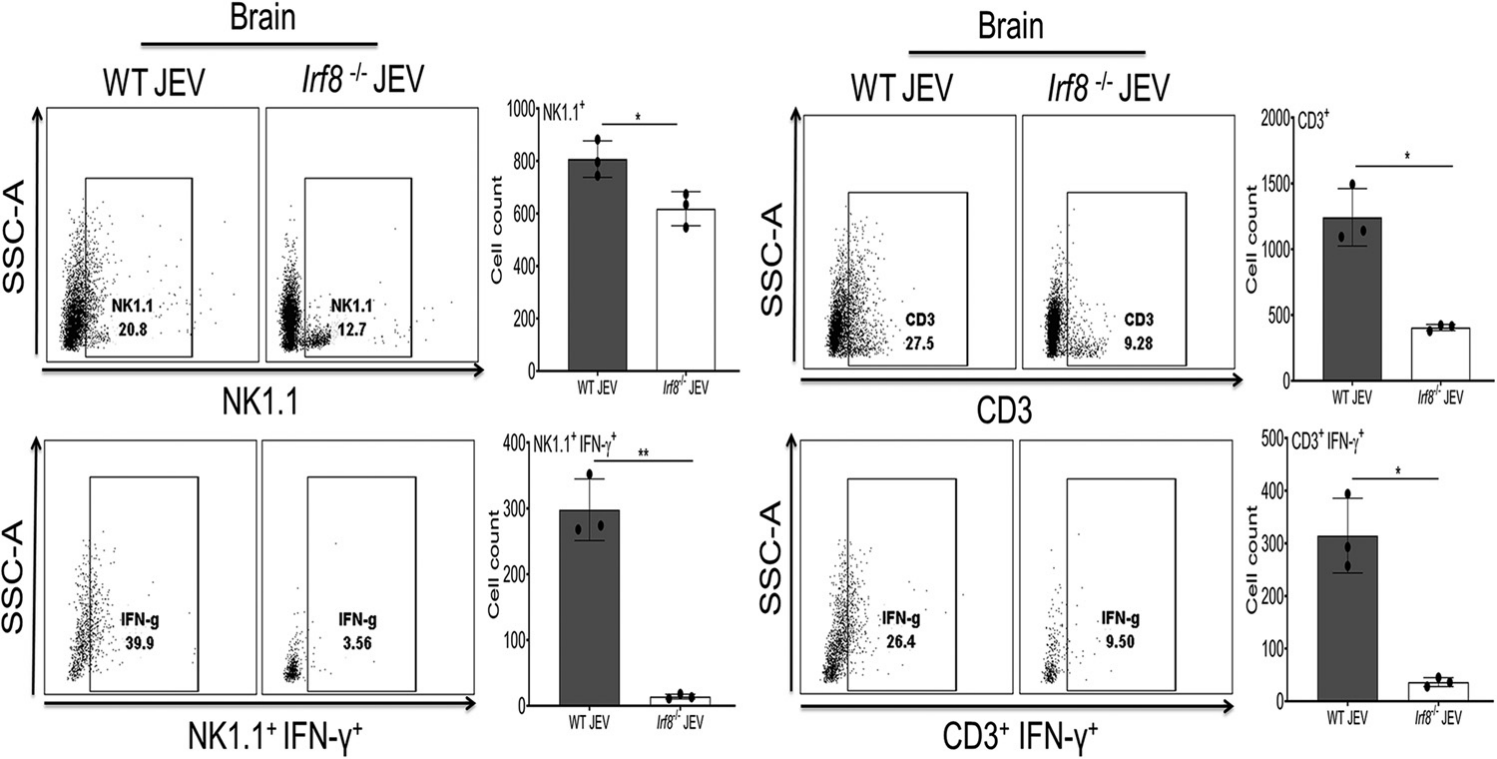
Intracellular staining of Ifnγ in infiltrating NK1.1^+^ and CD3^+^ cells in infected brains. Lymphocytes were enriched from the brains of WT (*n* = 3) and *Irf8^−/−^* (*n* = 3) mice from either the mock- or JEV-infected group by Percoll gradient for flow cytometric analysis. Representative dot plot (left) shows the percentage of cells in each quadrant, positive for the indicated markers on the axis. Bar graph (right) indicates the quantification of NK1.1^+^, NK1.1^+^ IFN-γ^+^, CD3^+^, and CD3^+^ IFN-γ^+^ expression on lymphocytes (**, *P* < 0.01; ***, *P* < 0.001; ****, *P* < 0.0001). Each dot in the bar graph represents cells from an individual mouse. Error bar represents mean ± SD.

Since *Irf8^−/−^* infected mice showed a lower *Ifnγ* level in the brain, we further checked the percentage of the *Ifnγ*-positive population in brain-infiltrating cells. The NK1.1^+^ and CD3^+^ cells are the primary immune cells that showed *Ifnγ^+^* staining in WT and *Irf8^−/−^* infected mice. Although NK1.1^+^ cells were abundant in WT and *Irf8^−/−^* infected mice, a very low percentage of NK1.1^+^ cells were positive for *Ifnγ* in *Irf8^−/−^* infected mice (Fig. 7). It has been reported previously that IRF8 deficiency increases the frequency of immature NK cells and decreases mature NK cells (21). Therefore, the reduced number of *Ifnγ^+^* NK1.1 infiltrating cells is probably a reflection of more immature NK cells in the *Irf8^−/−^* infected mouse brain. Irf8 also affects CD3^+^ T cell development (22). A lower *Ifnγ* level may be due to fewer CD3^+^ cells abundant in *Irf8^−/−^* infected mice (Fig. 5 and 6). Thus, reduced *Ifnγ* level in the *Irf8^−/−^* JEV-infected brain is probably due to a defect in the immune cell developmental process and lower abundance of T cells in infected mice.

### JEV replication augmented in *Irf8^−/−^* mice.

**FIG 8:**
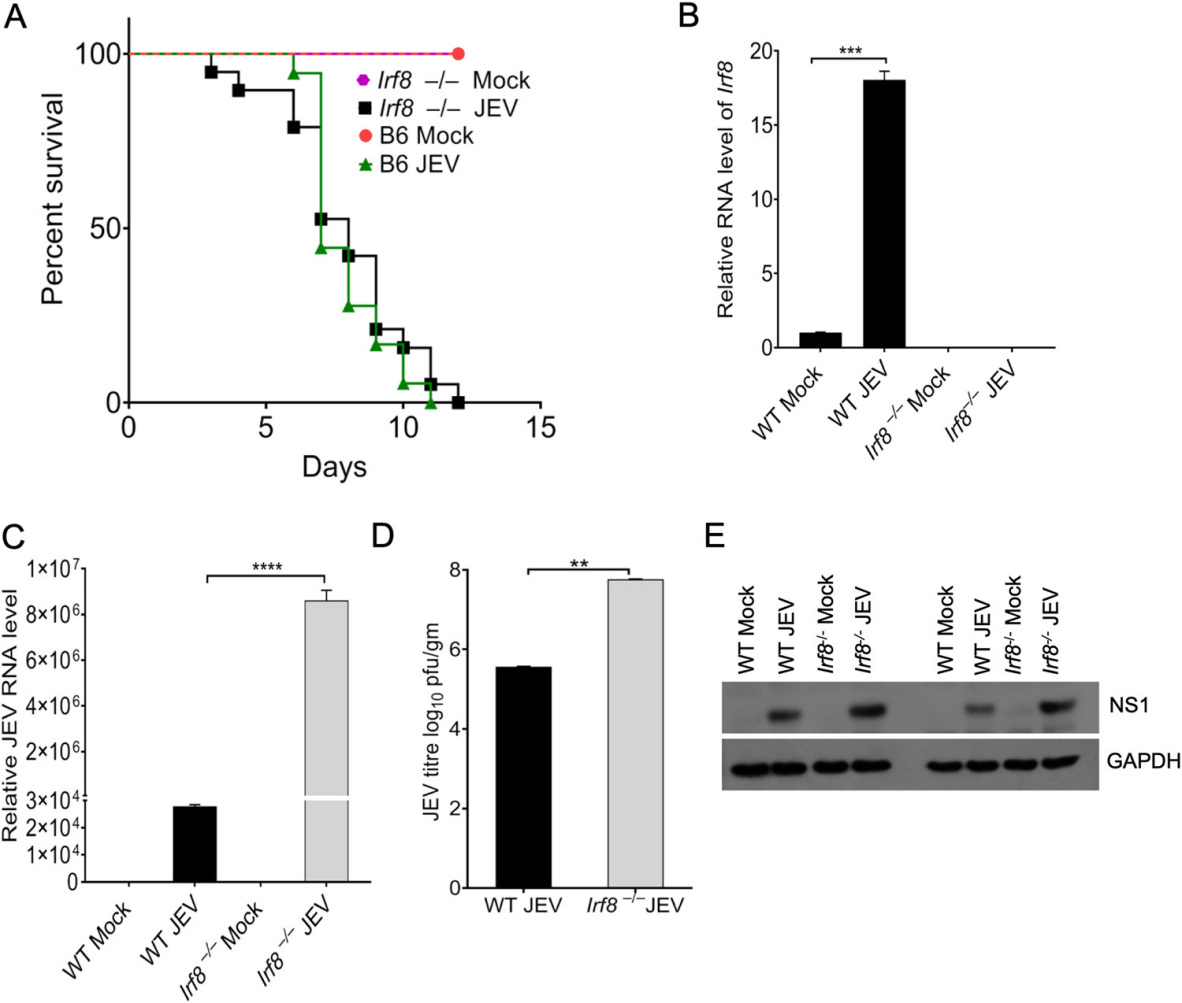
Effect of loss of IRF8 on JEV replication. (A) A survival study was performed to assess the survival of *Irf8^−/−^* mice to JEV infection. WT (C57BL/6) and *Irf8^−/−^* mice (*n* = 18 in each group) were mock or JEV (10^7^ PFU) infected and followed from the appearance of encephalitic signs until death; ****, *P* < 0.0001. Data shown are the combined results of three experiments. (B) The relative RNA level of *Irf8* transcripts was quantified by qRT-PCR in WT (C57BL/6) and *Irf8^−/−^* mice of mock- and JEV-infected RNA samples (normalized to *Gapdh* mRNA). (C, D) The relative level of viral and virus titer in the brain homogenates of infected WT and *Irf8^−/−^* mouse brains was determined through qRT-PCR and plaque assay. Virus titer is expressed as the number of virion particles per gram; **, *P* < 0.01. (D, C) ***, *P* < 0.001; ****, *P* < 0.0001. (E) The total protein of indicated groups was Western blotted for NS1 and GAPDH detection.

To assess the contribution of IRF8 to pathological inflammation, we infected *Irf8^−/−^* mice with the JEV P20778 strain. Virus replication in the brain, the appearance of neurological symptoms, and overall mouse survival were recorded over 15 days. While all C57BL/6 WT mice developed symptoms and succumbed by day 8 p.i., *Irf8^−/−^* mice developed severe symptoms early, but no significant differences were found in the survival time (Fig. 8A). All of the *Irf8^−/−^* mice were deficient in IRF8 at the transcriptional level (Fig. 8B). We checked JEV viral load and NS1 protein expression in WT and *Irf8^−/−^* infected mice to confirm that the mice died due to viral infection. We observed a significantly higher intracellular viral load and a higher NS1 protein expression level in *Irf8^−/−^* infected mice than in WT infected mice (Fig. 8C to E).

### Functional IRF8 expression in IRF8 KO cell line augments IFN-γ and reduced JEV replication.

**FIG 9:**
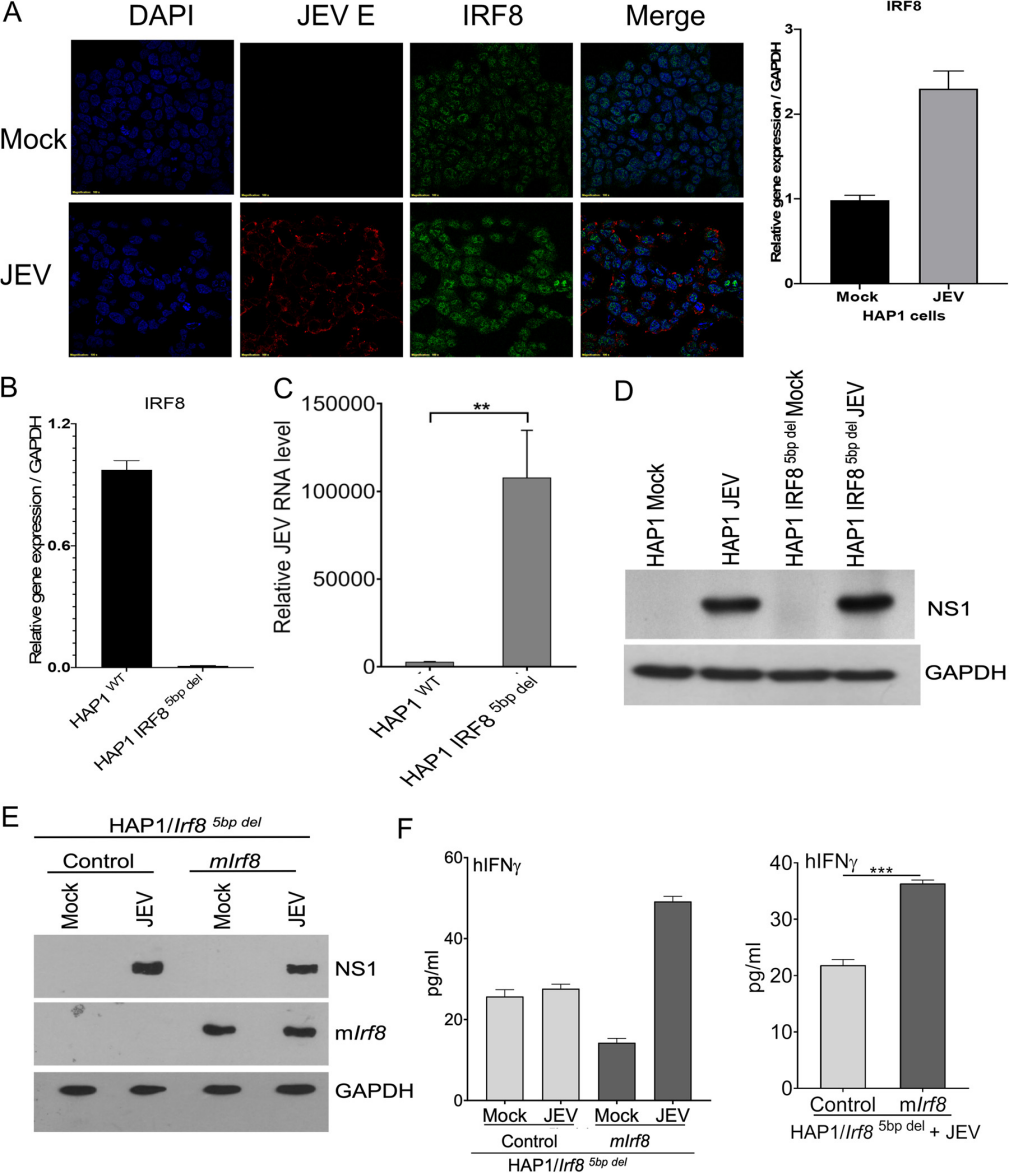
Effect of IRF8 overexpression in myeloid cell lines. (A) Haploid1 (HAP1) cell line was mock or JEV infected at an MOI of 5 for 24 h. Expression of IRF8 and viral envelope protein were detected by the immunofluorescence method. The relative level of human Irf8 transcript was quantified by qRT-PCR in a HAP1 cell that was mock or JEV infected at an MOI of 5 for 24 h (right). (B, C) The relative level of Irf8 and viral RNA were determined by qRT-PCR (**, *P* < 0.01). (D) Viral protein (NS1) was immunoblotted in the lysates of the mock and infected HAP1 and HAP1 *Irf8^5bp del^* cells. GAPDH was used as an internal control. (E) HAP1 *Irf8^5 bp del^* cells transduced with pMig-CD8t control or pMig-IRF8-cd8t plasmid were mock or JEV infected at an MOI of 5 for 24 h. Cell lysate was prepared from mock- and JEV-infected *HAP1 Irf8^5 bp del^* transduced with pMig-CD8t control or pMig-IRF8-cd8t plasmid, and Western blotting was performed to determine JEV and mouse IRF8 level in overexpressed cells. All qRT-PCR data are represented as mean ± SD and were normalized to *Gapdh*. (F) hIFN-γ expression was quantified in the protein lysates (left) and supernatant (right) of mock- and JEV-infected *HAP1 Irf8^5 bp del^* transduced with pMig-CD8t control or pMig-IRF8-cd8t plasmid groups using cytokine bead array kits analyzed by FCAP software. Representative data is shown; **, *P* < 0.01; ***, *P* < 0.001; ****, *P* < 0.0001.

Since IRF8 is expressed in myeloid lineage cells, we checked JEV infection in IRF8 knockout HAP-1 cells. JEV infects HAP-1 cells, as NS1 expression was observed in HAP-1 cells at 24 h p.i. IRF8 expression increased in JEV-infected HAP-1 cells compared to that in HAP-1 uninfected cells (Fig. 9A). To understand the effect of IRF8 on viral replication, we used IRF8 knockout HAP1 cells. These cells had a 5-bp deletion (*Irf8^5bp del^*) at the initiation site, leading to the generation of a truncated nonfunctional IRF8. The IRF8 at the transcript level is undetectable in *Irf8^5bp del^* cells. High viral load was also observed at RNA and protein levels in *Irf8^5bp del^* HAP-1 cells compared to those in the WT (Fig. 9B to D), indicating that the absence of IRF8 significantly augments JEV replication peripheral cells.

To understand if IRF8 works as a restriction factor for JEV replication, we overexpressed functional IRF8 in HAP-1 *Irf8^5bp del^* cells by transfecting the retrovirus vector containing functional mouse IRF8. After developing the stable cell line, we checked IRF8 and viral protein expression by Western blotting (Fig. 9E). JEV-NS1 protein level significantly decreased in HAP1 *Irf8^5bp del^* cells overexpressing mouse IRF8 protein (Fig. 9E). Further, we checked the IFN-γ level and found it increased upon JEV infection in cells and supernatant where functional IRF8 was present (Fig. 9F). Thus, our results point toward the association of IRF8 and IFN-γ, which together may affect viral replication.

## DISCUSSION

IRF8 plays a significant role in microglial activation and is also involved in mounting an antiviral response against invading pathogens. Microglial cells are susceptible to JEV infection, and prolonged microglial activation in the proinflammatory stage leads to severe inflammation in the brain, causing permanent neuronal damage. In this study, we have explored the possible role of IRF8 in the context of JEV replication and inflammation. Using the *Irf8^−/−^* mice, we demonstrated (i) the effect of IRF8 modulation on JEV replication in the brain, (ii) that the absence of the Irf8 function in *Irf8^−/−^* mice did not protect against JEV infection, (iii) that susceptibility to viral infection is also associated with the level of *Ifnγ*; and (iv) that the lack of *Ifnγ* or *Irf8* alters immune cell abundance in circulation and in the infected brain.

Viral infection in mice induces IRF8 expression in infected brains. Interestingly, the absence of functional IRF8 augmented viral replication in the infected mouse brain. Conversely, overexpression of functional IRF8 in the *Irf8*-defective cells (*Irf8^5bp del^*) attenuated viral replication. The increased viral replication may be because of reduced IFN-mediated antiviral response and was impaired in the *Irf8^−/−^* mice more than in WT mice. This is consistent with previous reports (21, 23, 24) where increased susceptibility to infections was reported in *Irf8^−/−^* mice. The absence of an adequate antiviral response may allow the virus to multiply.

Considering the high viral load in the brain, we expected *Irf8^−/−^* mice to die before the WT mice. However, we did not observe significant changes in survival time. It is important to note that JEV pathogenesis depends on viral load and the inflammation induced in the brain. In WT mice, an adequate antiviral response can probably restrict virus replication to some extent in the brain. As the virus enters the brain, prolonged microglial activation and increased infiltration of activated immune cells may cause severe neuronal damage, leading to fatal outcomes. Surprisingly, despite reduced microglia/macrophage proinflammation markers in the *Irf8^−/−^* mouse brain, the absence of IRF8 did not prevent the development of neurological signs nor its prolonged survival upon infection.

This is consistent with the previous report where *Irf8^−/−^* mice showed increased lethality following intranasal challenge with low-dose West Nile virus (WNV) compared with similarly infected WT mice (23).

It is important to note that although a high abundance of CD11b^+^ CD45^+^ cell populations were detected in the blood of *Irf8^−/−^* infected mice, less infiltration of these cells was observed in the infected brain (Fig. 5 and 6). This could be the reflection of reduced trafficking ability. Several studies demonstrated earlier that the expression of chemokines, such as *Ccl2*, *Ccl3*, *Ccl4*, *Ccl5*, and *Cxcl10*, during viral infection is associated with enhanced trafficking of leukocytes into the brain (25). Compared to WT mice, *Irf8^−/−^* mice exhibited a lower level of these chemokines in response to JEV infection (data not shown). Like West Nile virus disease (26), low expression of the chemokine receptor *Ccr2* expression in *Irf8^−/−^* and *Ifnγ^−/−^* mice in response to JEV infection probably indicates the reduced migration ability to infiltrate leukocytes in the inflamed brain. Our data are further supported, as shown in Fig. 2A, where reduced activation markers and cytokine expression were observed in response to viral infection in *Irf8^−/−^* mice.

It is important to note that although the *Ifnγ* level significantly goes down in *Irf8^−/−^* mice, TNF-α, IL-6, and MCP1 levels did not change and were comparable with those of WT mice. These data suggest that TNF-α, IL-6, and MCP1 are probably independent of IRF8 expression. It was reported earlier that the IRF8 expression was markedly upregulated in microglia but not in neurons or astrocytes (4). The neurons and astrocytes have been associated with producing inflammatory mediators in several neuroinflammatory scenarios, including viral infection in the brain (27). In the absence of microglial activation, astrocytes and neuronal cells may contribute to the production of TNF-α, IL-6, and MCP1 (16, 28–31). Another source of these cytokines could be the infiltrating neutrophils. The deficiency of IRF8 enhances neutrophil biogenesis (32). As a result, we observed a significant number of Ly6G^+^ cells infiltrated in the brain. It was reported earlier that these cytokines impart a detrimental effect on the pathology of JEV (16, 33). Therefore, the death of *Irf8^−/−^* mice may be due to a high abundance of proinflammatory cytokines, especially TNF-α, IL-6, and MCP1 in JEV-infected mouse brains.

We also observed that susceptibility to viral infection is associated with impaired production of *Ifnγ.* The *Ifnγ* production in response to viral infection reduced 3- to 4-fold in *Irf8^−/−^* mice. Similar to our study, Holtschke et al. reported an enhanced susceptibility to viral infections associated with impaired production of IFN-γ in *Irf8^−/−^* mice (34). Induction of IFN-γ appears to be critical for restricting JEV replication (35). It is important to note that *Ifnγ^−/−^* mice also exhibited a reduced level of *Irf8* and an increased viral load level in the mouse brain as observed in *Irf8^−/−^* mice. This is further supported by our *in vitro* complementation study (Fig. 9E and F), where the introduction of functional IRF8 into IRF8 KO cells enhanced IFN-γ expression concomitant reduction in viral replication. It was reported earlier that IFN-γ is critical in recovery from primary infection with JEV by a mechanism involving suppression of virus growth in the CNS and that T cells are the primary source of the cytokine that promotes JEV clearance from the brain (35). Moreover, the NK cell population is dispensable for control of JEV infection in the periphery and the CNS (35).

To examine whether reduced *Ifnγ* is associated with the abundance or defect in the function of immune cells, we checked immune cells in the blood and brain of *Irf8^−/−^* mice. As reported earlier, the transcription factor IRF8 is required for the development and maturation of myeloid cells (dendritic cells, monocytes, macrophages) (36, 37). We observed that the abundance of infiltrating immune cells in the infected brain directly reflects the cell population detected in circulation. Our FACS data from *Irf8^−/−^* mice showed reduced CD3^+^ T cells in the blood and the brain. Moreover, as shown in Fig. 7, for IRF8^−/−^ mice, reduced levels of Ifnγ^+^ cells are consistent with the reduced level of CD3^+^ T cells.

However, from our study, it is not clear how IRF8 regulates the expression of the IFN-γ gene. The regulatory regions of the IFN-γ do not have ISRE or a similar element. Therefore, the direct effect of IRF8 on gene transcription is unlikely. Similar to our study, Holtschke et al. (34) also reported that an association of IRF8 and IFN-γ expression is more likely indirect. It could reflect the influences of IRF8 on other elements of the cytokine network, which may be perturbed in *Irf8^−/−^* mice (34).

However, *Irf8^−/−^* or *Ifnγ^−/−^* mice exhibited reduced levels of activated microglia and infiltrated macrophage in brains, as most of the markers showed low expression in the brain (Fig. 2 and 5). The reduced expression level is possible because *Irf*8 is a critical transcription factor for maintaining microglia's physiological phenotype.

In conclusion, our study suggested that IRF8 modulation affects viral replication in the mouse brain. The absence of IRF8 alters immune cell abundance in the blood and brain of infected mice, reduces the IFN-γ level, and increases the viral load in the brain leading to death. Overall, our study provides evidence for the critical involvement of *Irf8* in immune cell abundance and antiviral response in the mice following JEV infection.

## MATERIALS AND METHODS

### Ethics statement.

The Animal Ethics Committee of the Translational Health Science and Technology Institute (THSTI) (approval number IAEC/THSTI/2017-8) and National Institute of Immunology (NII) (approval number IAEC/NII/494/18) approved all animal procedures that were used in the study. C57BL/6 WT, *Ifnγ^−/−^*, and *Irf8^−/−^* mice (3 to 4 weeks old) were maintained at the Small Animal Facility of the National Institute of Immunology, New Delhi. JEV-infected mice have been housed in HEPA filter-top cages under class II biohazard conditions, with food and water provided *ad libitum*. Virus propagation in pups and characterization was done at the Small Animal Facility available at the THSTI campus.

### Virus propagation.

We used JEV strain P20778 (JEV-S3) in all of the experiments. The virus (10^4^ PFU/10 μl) was inoculated in the cerebrum of 3-day-old suckling mice for propagation. The pups and mothers were housed and maintained at the Small Animal Facility within the NCR Biotech Science cluster campus. On day 3 p.i., brains were harvested from pups to prepare 40% (wt/vol) suspension by homogenizing the brain in minimal Eagle medium (MEM) (Invitrogen, Carlsbad, CA, USA). The homogenate was centrifuged to remove debris, and the supernatant containing the virus was aliquoted and stored at −80°C.

### Cell culture and viral infection.

The mouse microglia cell lines (BV2 and N9) (kindly provided by Anirban Basu, National Brain Research Centre, Manesar, India) were cultured in Dulbecco’s modified Eagle’s medium (DMEM) (Invitrogen, Carlsbad, CA, USA) and RPMI 1640 (HyClone; GE Healthcare Life Sciences, UT), respectively. Mouse neuronal cell line (N2a) was procured from the National Centre for Cell Sciences, Pune, India, propagated in Dulbecco’s modified Eagle’s medium (Invitrogen, Carlsbad, CA, USA). The human myeloid (HAP1 and HAP1 *Irf8^5bp del^* cell lines) cell lines (purchased from Horizon Discovery, Cambridge, UK; HZGHC004199c005) were maintained in Iscove’s modified Dulbecco medium (IMDM) (HyClone; GE Healthcare Life Sciences, UT). All media were supplemented with 10% fetal bovine serum (FBS) (Gibco, USA), 2 mM l-glutamine, and 100 μg/ml penicillin-streptomycin-glutamine (PSG) (Invitrogen, Carlsbad, CA, USA). Cell lines were maintained at 37°C in the CO_2_ incubator. Cells were seeded at 60% confluence and infected with JEV at a multiplicity of infection (MOI) of 5 for 1 h, following infection medium supplemented with FBS, and PSG was added. Cells were harvested at 24 h p.i. for lysate preparation in radioimmunoprecipitation assay (RIPA) buffer (Sigma-Aldrich, Saint Louis, MO, USA). TRIzol reagent (Invitrogen, Carlsbad, CA, USA) was added to cells for RNA extraction as per the manufacturer protocol.

### Mice and infection.

Mice (WT, *Irf8^−/−^*, and *Ifnγ^−/−^*) were obtained from The Jackson Laboratory, USA; C57BL/6 WT, *Ifng*^−/−^, and *Irf8*^−/−^ mice (3-4 weeks old) were maintained and infected at the Small Animal Facility of the National Institute of Immunology (NII), New Delhi. Mice of either sex were randomly divided into two groups. Mice in the infection group were inoculated through the intraperitoneal (i.p.) route with 1 × 10^7^ PFU/100 μl of JEV. In contrast, mice in the mock-infected group received an equal volume of sterile Eagle’s minimal essential medium (MEM) (Invitrogen, Carlsbad, CA, USA) by the same route. Mice were weighed and observed until they developed clinical symptoms of viral encephalitis. The time before the i.p. infection is referred to as day 0. For survival study, mice were followed until death. While preparing RNA or protein samples, mice were sacrificed 24 h after the onset of symptoms, followed by transcardial perfusion with ice-cold 1× phosphate-buffered saline (PBS). Brains were harvested and stored at −80°C until further use.

### Virus titration by plaque-forming assay.

Baby hamster kidney (BHK) cells obtained from the National Centre for Cell Science, Pune, India, were maintained at 37°C with 5% CO_2_ in minimal Eagle’s medium with 10% FBS, 1% penicillin-streptomycin, and glutamine. BHK cells with 60% confluence per well were used for titrating the virus. Cells were washed with 1× PBS followed by the addition of 10-fold serially diluted virus stock prepared in serum-free Eagle’s minimal essential medium for 1 h, incubated at 37°C with gentle rocking. Post incubation, cells were washed with 1× PBS and overlaid with 2 ml of agarose type VII (Sigma) in MEM, prepared by mixing a 1:1 ratio of 2× minimal Eagle’s medium (HiMedia) with 10% FBS and a 2% solution of low melting agarose. After 3 days, cells were fixed with 3.7% formaldehyde (Sigma). The overlay plugs were removed, and cells were stained with 1% crystal violet (Sigma) for 5 min. Plaques were counted, and the virus titer was calculated using the following formula: virus titer (PFU/ml) = average PFU/volume of infection (ml) × dilution factor.

### RNA extraction and qRT-PCR.

Total RNA was extracted from the brain using the RNeasy minikit (Qiagen, Hilden, Germany) following the manufacturer’s protocols. cDNA was prepared in two steps using the GoScript reverse transcription system (Promega, Madison, WI, USA). RNA extracted from samples was analyzed in triplicate by the real-time fluorescence detection method with SYBR green DNA binding fluorescent dye (SYBR Premix *Ex Taq*; TaKaRa, Otsu, Shiga, Japan) on a QuantStudio 6 flex real-time PCR (Applied Biosystems, Foster City, CA, USA) detection system. Relative expression was calculated using the threshold cycle (*C_T_*) method with uninfected as the reference, and *Gapdh* (glyceraldehyde-3-phosphate dehydrogenase) was used as an internal control. Microglial marker expression was quantified following the manufacturer's protocol for GeneQuery mouse microglial polarization markers quantitative PCR (qPCR) array kit (MGK114, Science cell, Carlsbad, CA, USA).

### Immunoblotting.

Mouse brains were minced and homogenized in Tris-HCl buffer (pH 7.5) with 1 mM phenylmethylsulfonyl fluoride (PMSF) (Sigma-Aldrich, Saint Louis, MO, USA). Samples were centrifuged at 13,000 rpm at 4°C for 15 min to obtain clear lysate that was aliquoted and stored at −80°C. According to the manufacturer's specification, protein concentration was quantified following bicinchoninic acid (BCA) assay (Pierce, Rockford, IL, USA). For Western blotting, an equal concentration of protein (30 μg) was resolved with 10% SDS-PAGE and transferred onto a nitrocellulose membrane. After blocking with 5% (wt/vol) skimmed milk, the membrane was probed with rabbit GAPDH antibody (at dilution 1:10,000, GTX100118; GeneTex, Irvine, CA, USA), in-house rabbit polyclonal antibody to JEV NS1 protein (at dilution 1:10,000), mouse JE1 antibody (at dilution 1:1,000, ab71671; Abcam, Cambridge, UK), rabbit Mannose receptor (at dilution 1:1,000, ab64693; Abcam, Cambridge, UK), mouse CD68 (at dilution 1:1,000, ab955; Abcam, Cambridge, UK), mouse IBA1 (at dilution 1:1,000, MABN92; Merck Millipore, Darmstadt, Germany), rabbit STAT1 (at dilution 1:1,000, sc346; Santa Cruz Biotechnology, Dallas, TX, USA), rabbit Phospho-STAT1 (at dilution 1:1,000, Tyr701-58D6; Cell Signaling Technology, MA, USA), rabbit Phospho-STAT2 (at dilution 1:1,000, Y690-D3P2P; Cell Signaling Technology, MA, USA), rabbit STAT2 (at dilution 1:1,000, D9J7L; Cell Signaling Technology, MA, USA), mouse STAT3 (at dilution 1:1,000, 124H6; Cell Signaling Technology, MA, USA), rabbit Phospho-STAT3 (at dilution 1:1,000, 124H6; Cell Signaling Technology, MA, USA), and mouse IRF8 antibody (at dilution 1:1,000, sc365042; Santa Cruz Biotechnology, Dallas, TX, USA) to check their expression in infected and uninfected mouse brain lysate. For chemiluminescence detection, a luminol reagent (Santa Cruz Biotechnology, Dallas, TX, USA) was used, and the signal was visualized in the ChemiDoc XRS system (Bio-Rad, Hercules, CA, USA). Densiometric analysis of the blot was done using ImageJ software to determine the signal intensity.

### Cytokine bead array.

Cytokine bead array (CBA) was performed to quantitatively measure the cytokine level of six cytokines (IL-6, TNF-α, IFN-γ, MCP1, IL12p70, IL-10) using an assay kit procured from BD Biosciences (San Diego, CA, USA). Four other cytokines (TGF-β, IL-27, IL-23, and IL-1β) were measured using an assay kit procured from BioLegend (San Diego, CA, USA), and data were analyzed using CBA software (FCAP 3.0.1 and Qognit). Twenty-five micrograms of protein lysate and 100 μl of cell supernatant were used for analysis. Data were acquired using CellQuest Pro software in the FACSCalibur cell analyzer (BD Biosciences, San Jose, CA, USA). The quantity of the cytokines detected in the lysate samples was measured against the standard curve obtained from the defined concentration of protein provided with the assay kit.

### Immunostaining.

Mice were perfused with sterile cold 1× PBS as well as with 4% paraformaldehyde (PFA). The brains were removed and kept in 4% paraformaldehyde for 24 h at 4°C followed by incubation in 30% sucrose solution until immersion. Brains were taken out and embedded in OCT (Tissue-Tek, Saint Louis, MO, USA) and frozen at −20°C. Thirty-micrometer cryostat sections of the brain were made with the help of cryostat (Microm HM550; Thermo scientific, Runcorn, UK) and mounted on slides. The antigen retrieval process was performed at 70°C using antigen unmasking solution (Vector Laboratories, Burlingame, CA, USA). After permeabilization and blocking, the slides were processed for the JEV NS1 antigen. Images were captured at ×60 magnification using a confocal microscope, Olympus FV3000. For *in vitro* immunostaining, HAP1 cell lines seeded at 60% confluence were either mock and JEV infected (MOI = 5) for 1 h, then inoculum was removed, and complete medium was added to the cells. Cells were washed with 1× PBS and fixed with 4% PFA (Sigma-Aldrich, Saint Louis, MO, USA). After permeabilization and blocking, cells were incubated with the IRF8 antibody (at dilution 1:500, ab28696; Abcam, Cambridge, UK) as well as NS1 (1:1,000) for overnight incubation at 4°C. The fluorochrome-conjugated secondary antibodies (A11011, Alexa fluor 568 goat anti-rabbit; A11029, Alexa fluor 488 goat anti-mouse; Invitrogen; 1:500) were used to detect the proteins. The slides were observed under a confocal microscope (Olympus FV3000) at ×100 magnification. Images were captured and analyzed using software FluoView (FV 31S-SW) and cellSens Dimension, respectively.

### Immune cell isolation from mouse blood and brain for flow cytometry analysis.

Blood was collected from mice through retro-orbital bleeding and added to ACK lysis buffer (BioLegend, WaySan Diego, CA) for red blood cell lysis as per manufacturer protocol. Mice were anesthetized with ketamine and xylazine and perfused with ice-cold PBS. The brain was removed and homogenized with a Dounce homogenizer in cold Hanks balanced salt solution (HBSS) buffer (Sigma-Aldrich, Saint Louis, MO, USA). Brain homogenate was resuspended to prepare 30% isotonic Percoll (Sigma-Aldrich, Saint Louis, MO, USA), which was overlayered on 70% isotonic Percoll. Then a gradient was centrifuged at 500 × *g* for 25 min at 18°C. Mononuclear cells were collected from the 30%/70% interface and washed with PBS. To identify microglia, monocytes, and granulocytes, isolated mononuclear cells from blood and brain were first preincubated with anti-mouse CD16/32 antibody TruStain FcX as per manufacturer protocol (BioLegend, San Diego, CA) for 15 min at 4°C to block Fc receptors and then simultaneously stained with fluorochrome-conjugated antibodies (APC-CD11b, FITC-CD45 [at dilution 1:100; Miltenyi Biotec, Gladbach, Germany], FITC-Ly6C [at dilution 1:50; Invitrogen, Vilnius, Lithuania], V450-Ly6G [at dilution 1:50; BioLegend, San Diego, CA, USA], BV421-IFN-γ [at dilution 1:25; BioLegend, San Diego, CA, USA]). For T cell immune profiling, all of the antibodies were procured from BioLegend (BV510 anti-mouse CD3ε [at dilution 1.5:50], PerCP/Cyanine5.5 anti-mouse CD4 [at dilution 1:50], Brilliant Violet 421 anti-mouse CD8a [at dilution 1:50], and APC/Cyanine7 anti-mouse NK-1.1 [at dilution 1:25]). Cells were then rinsed with FACS buffer and run on the BD FACS Verse (BD Biosciences, San Jose, CA). For intracellular staining, cells were fixed and permeabilized using BD Cytofix/Cytoperm (BD Biosciences, San Jose, CA) as per the manufacturer protocol. Data were analyzed using BD FACS Suite v1.0.6 (BD Biosciences, San Jose, CA) and FlowJo v10 (FlowJo LLC).

### Transduction of haploid cells.

Packaging cells were transfected with retroviral vectors pMig-control-IRES-hCD8t and pMig-Irf8-IRES-hCD8t (38, 39), and supernatant containing virus was collected after 48 h. For retroviral transduction, HapI-Irf8^5bp del^ cells (1 × 10^6^) were incubated with Mig-control-IRES-hCD8t and Mig-Irf8-IRES-hCD8t supernatants of retroviruses expressing the truncated human CD8 surface marker by spinoculation (2,400 rpm, 33°C, 1 h) with 4 μg/ml Polybrene. One day postransduction, cell populations were analyzed by flow cytometry using an anti-human CD8-PE antibody (BioLegend, San Diego, CA, USA), and the human truncated CD8-expressing population was purified by magnet-activated cell separation using biotin-labeled anti-human CD8α antibody from BD Biosciences and streptavidin microbeads from Miltenyi Biotec (40). Sorted cells were infected with JEV at an MOI of 5 and harvested after 24 h of infection.

### RNA sequencing library preparation.

According to the manufacturer’s protocol, total RNA was extracted using the RNeasy kit with on-column DNase treatment (74004; Qiagen). RNA quality was assessed using an Agilent TapeStation (Agilent, Palo Alto, CA, USA), and RNA concentration was quantified by Qubit 4.0 spectrophotometer. Total RNA was used as the input material for rRNA depletion and RNA sequencing library preparation. The library was prepared for sequencing using the TruSeq standard total RNA library preparation kit (Illumina, San Diego, CA, USA). Each sample was prepared using 300 ng of total RNA. The resulting cDNA libraries were quantified using the KAPA library quantification kit (KAPA Biosystems, Wilmington, MA, USA), and quality and size were checked using the Agilent high-sensitivity DNA kit. The average fragment length of the cDNA libraries was 330 bp. RNA-Seq libraries were sequenced using paired-end 100-base (PE100) sequencing chemistry on NovaSeq instruments following the manufacturer’s protocols (Illumina, San Diego, CA, USA). RNA sequencing was performed at the sequencing core facility (Core Technologies Research Initiative [CoTeRI]) of the National Institute of Biomedical Genomics (NIBMG) in Kalyani, India.

### RNA-Seq data analysis.

The raw reads corresponding to Illumina RNA-Seq FASTQ files were aligned in Strand NGS software v3.4 against the mouse reference genome (Build mm10, gene feature: ensemble genes, library layout: paired-end). Quantification was done to obtain the read densities using Strand NGS software. Differential expression analyses of mRNA from different groups were performed using the DESeq script II via the script editor in Strand NGS with a *P* value of ≤0.05 and a fold change of 2.0-fold.

### Statistical analysis.

Data were analyzed using GraphPad PRISM 8.0 (CA, USA) and presented as mean ± standard deviation (SD) of at least three independent experiments with three replicates. Statistical significance was determined using Student's *t* test, one-way analysis of variance (ANOVA), two-way ANOVA, and Log rank (Mantel-Cox) test. A *P* value of <0.05 was considered statistically significant.

### Data availability.

The data discussed in this publication have been deposited in NCBI's Gene Expression Omnibus (41) and are accessible through GEO series accession number GSE154002.
